# Role of Metformin in Polycystic Ovary Syndrome (PCOS)-Related Infertility

**DOI:** 10.7759/cureus.44493

**Published:** 2023-08-31

**Authors:** Ghalia M Attia, May M Almouteri, Fatimah T Alnakhli

**Affiliations:** 1 Medical Histology and Cell Biology, Faculty of Medicine, Mansoura University, Mansoura, EGY; 2 Medicine and Surgery, Taibah University, Medina, SAU

**Keywords:** polycystic ovary syndrom, insulin resistance, hyperandrogenism, infertility, metformin

## Abstract

Polycystic ovarian syndrome (PCOS) is considered the most prevalent endocrinological disorder, which affects some women and it is characterized by anovulation and hyperandrogenism, with morphologic changes in the ovary, inappropriate gonadotropin secretion, and insulin resistance (IR) with accompanying compensatory hyperinsulinemia. PCOS was associated with some degree of IR which probably contributes to hyperandrogenism. Many studies showed that metformin, when used to treat PCOS, significantly reduced serum androgen levels, improved insulin sensitivity, restored menstrual cyclicity, and was successful in triggering ovulation. As a result, metformin may be useful in treating PCOS-related infertility. The aim of this review was to clarify PCOS, its prevalence, particularly in Saudi Arabia, its pathogenesis, its impact on the patient's health, and to explain the uses of metformin, its mechanism of action, and its role in the treatment of PCOS-related infertility.

## Introduction and background

Polycystic ovarian syndrome (PCOS) is the most widespread endocrinological condition and affects roughly 4%-12% of women [1]. It is proven to be the main cause of infertility in women [2]. There are some clinical characteristics of PCOS, which are menstrual irregularity with associated anovulatory infertility and hyperandrogenism [3]. Over time it became obvious that the syndrome was quite heterogeneous with a wide clinical spectrum. It is considered a syndrome, not a disease. Hirsutism was reported to occur in about 69% of affected females, infertility in 74%, obesity in about 40%, amenorrhea in slightly over 50%, functional bleeding in 29%, and cyclical menses in 12%, based on about 1,080 cases from the literature are collected [3].

A significant association between the level of insulin and testosterone was observed in PCOS. It was obvious that IR is a popular feature of the disorder and is not relevant to obesity. The resistance of insulin is generally known as "a condition of the tissue, a cell, also an organism where a higher amount of insulin than usual is required to grab quantitatively regular response'' and keep the level of glucose within the regular range. Approximately 50%-70% of PCOS women have IR, which has a critical role in PCOS pathogenesis [4-5]. As a result of the increase in circulating insulin levels in the ovary, it is thought that the insulin level in the circulating system when it exceeds the regular level, contributes to both redundant anovulation and redundant androgen production [6].

Clomifene citrate (CC), metformin, and thiazolidinediones [thiazolidinediones (TZDs), e.g., rosiglitazone and pioglitazone] which are often used to increase insulin sensitivity and decrease IR are considered to be the first line ovulation-inducing drugs in infertile women with PCOS either alone or in combination [7-9].

Metformin is a drug that is commonly utilized orally as an antihyperglycemic agent and is confirmed as a treatment for type 2 diabetes mellitus (DM) by the US Food and Drug Administration (FDA). The benefits of metformin on insulin sensitivity have been shown in women with PCOS who are not DM. Menstrual cyclicity is increased as a result of the use of metformin, which also enhances the decrease in androgen circulation and ovulation. Weight loss is considered a factor that influences metabolic advantages [10]. The rationale for this work is the widespread of PCOS among young girls and its associated impact on infertility with social and marital problems, therefore, we want to throw light on this problem and discuss how to manage it.

The information in this review was collected from many high-quality papers that are published on many websites like PubMed and Science Direct.

## Review

What is PCOS? 

Polycystic ovarian syndrome (PCOS) is the most widespread endocrinological condition which is characterized by anovulation, hyperandrogenism, and infertility, with irregular menstrual cycles as a clinical manifestation. It was found to depend on many factors; complex genetic, heterogeneous, and endocrinal disorder [11].

The cause of PCOS is poorly understood, and both the diagnosis and treatment of the disorder are controversial. Women with this syndrome have morphological changes in ovarian (polycystic), abnormal gonadotropin secretion, and IR with associated compensatory hyperinsulinemia [12].

Recently proposed that the syndrome may be diagnosed after excluding other medical conditions that cause irregular menstrual cycles and androgen excess, and deciding that it exists in a minimum of two of the following: anovulation or oligoovulation (usually appeared as amenorrhea or oligomenorrhea), clinical manifestations in androgen excess, and high levels in circulating androgens (hyperandrogenemia) and polycystic ovaries as defined by ultrasonography. Females with PCOS have a certain deflection of gonadotropin secreting almost always in comparison to females with regular menstrual cycles. The most common form of chronic anovulation is oligomenorrhea (lower than nine men each year) or amenorrhea [12]. 

Anovulation cycles might have a decreased fertility, and result in dysfunctional uterine bleeding, in addition to cutaneous manifestations of hyperandrogenemia in the PCOS such as acne, male-pattern hair loss (androgenic alopecia), hirsutism, and Acanthosis nigricans which is considered as a hyperinsulinemia cutaneous marker. A small proportion of females with the condition of the polycystic ovary are overweight; others are obese, some highly so. While obesity itself is not considered the initiating event in the development of the syndrome, it may worsen the related excess adiposity metabolic and reproductive disturbances [13].

Prevalence of PCOS

The prevalence of PCOS is exactly unknown in Saudi Arabia. On studying the prevalence of PCOS in young unmarried female students of Almadinah Almunawwarah Taibah University, who were showing menstrual irregularities between January 2012 and December 2012. From a cohort of 201 participants, it was found that 108 (53.7%) were diagnosed with PCOS with a mean age of 21 ± 2 years. Menstrual irregularities, demographic details, and dermatological manifestations were reported in 108 cases of PCOS. In 97 (89.8%) patients, there were 12 or more follicles measuring 2-9 mm, followed by 89 (82.8%) patients with peripheral distribution of ovarian follicles. The estimated high prevalence of 53.7% of the cases examined may be explained by the high incidence of obesity in Saudi Arabia associated with PCOS [14]. A study conducted on 29-43-year-old Saudi female patients with a suggested diagnosis of PCOS illustrated the prevalence of 64.5% obese and 24.2% overweight cases [15].

On studying the characteristics, phenotypes, and prevalence of polycystic syndrome, it was proven that it is a common disorder among reproductive-age women, which represents from 4% to 21%. The prevalence of PCOS was determined by diagnostic criteria approximately 4%-6.6% based on NIH 1990 standards. In addition, about 4%-21% based on the Rotterdam standard was applied in 2003. Even though these restrictions of released prevalence studies are proper to sample and outcome definition, PCOS prevalence by NIH 1990 standard is still almost steady. Over the last 10 years, the description of the syndrome has been improved [16]. 

Although some signs of progression were observed in comprehending the PCOS phenotype within female teenagers and post- and peri-menopausal females, more studies are needed. The latest evidence indicates considerable differences in phenotype, morbidity, and ethnicity among PCOS females known in the clinical setting against the overall population. Many epidemiologic statements are needed among medically unbiased PCOS populations to understand this syndrome's natural course better [16].

Pathophysiology of PCOS**

No reason has been elicited for PCOS. Instead, it is expected to be a syndrome related to the combination of environmental factors and genetics [17]. Immunohistochemical studies of theca cells from females with PCOS showed luteinizing hormone (LH) receptors and steroidogenic enzyme overexpression, as well as cytochrome P450c17 enzyme [18].

During puberty, the hypothalamic-pituitary-ovarian axis is matured and the circulating LH levels are subsequently increased [19]. In particular, adolescents with PCOS show increased frequency and amplitude of gonadotropin-releasing hormone (GnRH) and LH pulses, as well as an increased ratio of LH to follicle-stimulating hormone (FSH) [20]. Moreover, insulin, also, acts as an essential part of the regulation of androgen. As insulin gets high, there is going to be a mutual decline in the level of the serum sex hormone binding globulin (SHBG) via inhibiting the liver's form of production of these hormones [19]. Many studies have found that hyperinsulinemia and IR are important outcomes in females suffering from PCOS [21].

Resistance to insulin induces compensatory hyperinsulinemia that drives some of the phenotypic PCOS characteristics [22]. Laboratory research has shown that LH and GnRH secretions are elevated in response to the infusion of insulin [23]. In addition, it can be active in response to LH stimulation as well, which amplifies steroidogenesis in all granulosa cells and ovarian theca that stimulates ovarian hyperandrogenism by stimulation of 17α-hydroxylase activity in theca cells [24]. Hyperinsulinemia also intensifies insulin growth factor-1 (IGF-1)-stimulated and LH-stimulated androgen production [25].

The central paradox in the pathophysiologic association between hyperinsulinemia and hyperandrogenemia in PCOS is that the ovary remains sensitive to insulin activity and subsequent androgen production, despite a systemic state of IR, setting the stage for the “selective insulin resistance” theory [22].

During the development of ovarian follicles, a group of primordial follicles grows into primary, then into secondary follicles with the appearance of antrum in these follicles of which one large antral follicle develops and called a mature Graaffian follicle is selected to ovulate.

Therefore, folliculogenesis requires multiple endocrine and intraovarian paracrine interactions for appropriate oocyte development. During PCOS, there may be disruption of the development of follicles when IR, hyperinsulinemia, ovarian hyperandrogenism, and altered intraovarian paracrine signaling happen. Therefore, follicular arrest in patients with PCOS is accompanied by anovulatory subfertility, small antral follicle aggregation within the periphery of the ovary, and menstrual irregularity, offering polycystic morphology [26].

Adiposity was related to menstrual dysfunction, and concentrations of androgen [18]. Obesity induces the resistance of insulin and exacerbates the hyperandrogenism found in PCOS [20]. Excessive adiposity can lead to excess androgen, as the adipose tissue contains several steroid enzymes that transform androstenedione into testosterone, and testosterone into more potent androgen, dihydrotestosterone (DHT). Where obesity is present, IR is increased [23].

Recently it was suggested that genetic causes have been involved in PCOS pathophysiology, with specific emphasis on genes influencing reproductive hormone biosynthesis and function, chronic inflammation, and cell metabolism. Several fundamental genes related to steroidogenesis are implicated, which are CYP19, CYP17A1, HSD17B6, HSD17B5, and CYP21 [27].

Impact of PCOS

Polycystic ovarian syndrome has a significant negative effect on the body's physiology and metabolism because it can develop into a metabolic syndrome (MS) with IR, hyperinsulinemia, hypertension, dyslipidemia, and abdominal obesity. This syndrome manifests as common metabolic traits and leads to serious long-term consequences, including type 2 diabetes mellitus, cardiovascular disease, and endometrial hyperplasia [27].

Infertility

One of the most common causes of infertility is PCOS, which is associated with an increased risk of miscarriage after either an unplanned pregnancy or a pregnancy with assistance, and the occurrence of ovarian hyperstimulation syndrome (OHSS) in assisted pregnancy. Women with polycystic ovaries have been proven to have a high incidence of spontaneous abortions in the first trimester (25%-73%). It is noted that 81% of women with frequent fetal loss have defects in LH secretion, and among those with recurrent miscarriages, both with and without PCOS elevated androgen levels have been reported. Anovulatory infertility, in PCOS due to arrested folliculogenesis, is frequently associated with obesity and IR [28].

Metabolic Dysfunction

The MS, also known as insulin resistance syndrome or syndrome X, indicates a group of many cardiovascular risk factors, including hyperinsulinemia, IR, hypertension, atherogenic dyslipidemia, and abdominal obesity, as recurrent metabolic features. MS is increasingly relevant to the risk of cardiovascular disease and type 2 diabetes. One of the most common metabolic abnormalities in PCOS patients is IR (71%) followed by obesity (52%) and dyslipidemia (46.3%), with an incidence of 31.5% for MS. Abdominal obesity is the main clinical feature of MS or cardiovascular dysmetabolic syndrome, while inactivity and atherogenic diet are the major risk factors [28].

Insulin Resistance

Insulin resistance plays a main role in PCOS pathogenesis It has a multifactorial pathogenesis and has been associated with components of MS, such as cardiovascular risk, endothelial dysfunction, and hypertension, which is conceived as the primary stage of the atherosclerosis process and a shorter lifespan. Anovulatory women with PCOS compared with weight-matched control participants are relatively hyperinsulinemic and more insulin-resistant. Approximately 50%-70% of all women with PCOS have some degree of IR, and this hormone insensitivity is likely to lead to the hyperandrogenism that is responsible for PCOS signs and symptoms. Moreover, the strong finding that has been proved in some ethnic groups that were used in many studies is the link between increased IR and PCOS which contributed to the hyperandrogenism that is responsible for the PCOS signs and symptoms [15].

Hyperinsulinemia

Hyperinsulinemia can result from IR, inherent abnormalities in insulin synthesis/secretion, and obesity, whether because of inherent defects in insulin action or by weight gain, activates both adrenal and ovarian cytochrome P450c17a activity and may clarify how early adrenarche during pubertal gonadotropin activation leads to PCOS in girls [15].

By peripherally inhibiting hepatic synthesis, hyperinsulinemia decreases serum sex hormone binding globulin (SHBG), favoring free circulating androgens, and decreasing insulin-like growth factor binding protein-1 (IGFBP-1), allowing more IGF-1 to be accessible locally and peripherally. Additionally, it increases LH pulse amplitude or potentiates the effects of LH on ovarian steroidogenesis, inducing hyperandrogenism. Moreover, long-term hyperinsulinemia as in the case of PCOS stimulates leptin secretion from adipose tissue, the elevated levels of which have been associated with adverse effects on reproductive function [28].

Metformin and its role in PCOS-associated infertility

Uses of Metformin

Metformin also called dimethyl biguanide is an oral antidiabetic drug. It helps restore insulin response in the body, and also it decreases the amount of liver glucose production that the intestines or stomach can absorb [29]. The usage of metformin in the past was to stop diabetes existing for people who have a higher risk of having it. Another function was to use it by females who have the syndrome of PCOS. Moreover, metformin can make menstrual cycles more regular and improve fertility [30]. It is now commonly prescribed as an anti-diabetic drug; however, significant concerns have been posed about its adverse effects, especially ketoacidosis [31].

Mechanism of Action of Metformin in PCOS

The use of metformin is related to ovulation and increased menstrual cyclicity, in women with PCOS. Its principal clinical function is to inhibit the production of hepatic glucose, increasing insulin sensitivity in peripheral tissues and decreasing intestinal glucose uptake [30]. Metformin also results in the reduction of fasting serum insulin (thus the risk of hypoglycemia is minimal) by about 40% and leads to a reduction in the mean weight by 5.8% [32]. Additionally, it inhibits mitochondrial respiratory chain complex I (mitochondrial stress) by interfering with cellular respiration, which imitates a cell starvation condition by decreasing intracellular ATP. Accordingly, the adenosine monophosphate (AMP) pool increases, activating the AMP-activated protein kinase (AMPK), which is a cell energy sensor [33].

The AMPK is directly activated by AMP molecules through binding to the gamma-subunit of the enzyme or indirectly through the inhibition of its dephosphorylation. The AMPK stimulation also increases the translocation of glucose transporters (GLUT) to the cell membrane and increases the transport of glucose into the cell (increased use of glucose) [34].

The activation of AMPK also results in a reduction in the activity of acetyl-CoA carboxylase (ACC) which in turn reduces oxidation of fatty acids and suppresses lipogenic enzymes. Suppression of fatty acid oxidation results in decreased hypertriglyceridemia, thus reducing the availability of energy for gluconeogenesis. This is linked with increased clearance of very low-density lipoprotein (VLDL) and decreased synthesis. Lipolysis inhibition and reduction in triglyceride levels will lead to increased insulin sensitivity through reduced lipotoxicity [35].

Metformin and Ovulation Induction

Obesity significantly affects both natural and assisted conception, as well as the possibility of a healthy pregnancy. Greater IR is associated with increasing obesity. Metformin appears to have direct effects on ovarian function in addition to inhibiting the production of hepatic glucose and improving cellular insulin sensitivity. Therefore, it makes sense to think that medications like metformin that lower insulin and make the body more sensitive to insulin would help with the symptoms and the results of pregnancy for PCOS women [36].

Most of the initial studies on metformin that searched for its role in the management of PCOS were observational. Most of the research indicated that metformin had a substantial impact on restoring menstrual cyclicity compared with placebo, decreased serum androgen levels, and was successful in induction of ovulation either alone or in conjunction with clomifene [37]. Nevertheless, those early promising results were not substantiated by subsequent larger randomized trials. However, some research indicated that metformin therapy could produce a weight reduction [38]. This was not supported by the large randomized controlled trials and systematic reviews [39].

Metformin appears to be less effective in patients who are significantly obese and whose body mass index (BMI) is greater than 35 kg/m2 [40]. However, there is no agreement on predictors for response or the appropriate dosage of metformin and whether its dose should be modified or adjusted for body weight or other variables. Doses ranging from 500 to 3000 mg/day are used and the most common dosage regimens are about 500 mg three times daily or 850 mg twice daily. Metformin seems to be safe in pregnancy, while customary advice is to avoid it once pregnancy occurs. There is no clear evidence to indicate that metformin decreases the risk of either abortion or gestational diabetes [40].

Some 143 anovulatory women in the United Kingdom with a mean BMI of 38 kg/m2 were evaluated in the biggest prospective, randomized, double-blind, placebo-controlled research trial to determine the cumulative effects of lifestyle modification and metformin (850 mg twice a day). In order to establish a realistic goal that could be attained with an average reduction of 500 kcal per day in energy consumption, each participant underwent an individualized assessment by a dietitian. As a result, both the metformin-treated group and the placebo group were able to lose weight, however, the amount of weight loss was the same for both groups. Menstrual cyclicity was shown to be more prevalent in women who lost weight [40].

In one study involving 228 women with PCOS, three women were left out due to medical problems, 111 women were treated with CC plus metformin (metformin group) and 114 were allocated to CC plus placebo (placebo group). The ovulation rate in the metformin group was 64% compared with 72% in the placebo group. There was no significant difference in either the rate of ongoing pregnancy (40% vs. 46%) or the rate of spontaneous abortion (12% vs. 11%). Moreover, a significantly larger proportion of women in the metformin group discontinued treatment because of side effects [40]. In another study, 676 infertile women with PCOS were randomized to multiple treatment arms including metformin 1 g twice daily + placebo, CC + placebo, or metformin + CC. Women were studied for six cycles or 30 weeks and the medication was discontinued when pregnancy occurred. It was proved that live birth rates were 7.2% (15/208), 23% (47/209), and 26.8% (56/209), respectively [41]. Thus, it was concluded that metformin in combination with CC could be somewhat successful in the treatment of women who are anovulatory and infertile with PCOS [42].

Metformin and In Vitro Fertilization

There was conflicting evidence regarding how metformin affects egg production, oocyte or embryo quality, or both. Metformin and placebo were compared in nine trials before or after assisted reproductive technology cycles in a Cochrane review with 816 women. In the metformin group, clinical pregnancy rates have increased. However, the number of events for the pregnancy rates dropped from 775 to 551 for the live birth rates, which may have weakened the power of the meta-analysis with regard to live birth rates. The miscarriage rate remained unaffected. The risk of OHSS was significantly reduced in women given metformin when a long gonadotrophin-releasing hormone (GnRH) agonist protocol was used. Additionally, by modulating the ovarian response to stimulation, the risk of OHSS in these patients decreased. However, the short GnRH antagonist protocol is currently recommended for women at risk from OHSS, for whom metformin's function is unclear [43].

Metformin and Pregnancy

Treatment with metformin should be stopped once pregnancy is confirmed, whether used as a first-line treatment or in women who are clomifene-resistant, as it is not authorized for use in pregnancy. However, there is no evidence of metformin teratogenicity in animal or human fetuses. For instance, pregnant women with type 2 diabetes mellitus who were treated with metformin showed no rise. In the occurrence of significant congenital abnormalities when compared with untreated type 2 diabetes pregnant women [44]. Metformin treatment has been recommended to minimize miscarriage rates in women with PCOS, although there is debate over the basis of the correlation between miscarriage and PCOS.

There was evidence that supported that metformin can reduce miscarriage rates. In a retrospective study that included many women with polycystic ovaries, metformin was proven to reduce spontaneous miscarriages in the first trimester; the reduction in the risk of gestational diabetes in women with PCOS has also been shown in metformin therapy. In a retrospective study, about 10-fold decrease in gestational diabetes development in non-diabetic women with PCOS was consistent with the use of metformin during pregnancy [45].

Dose and regimen for metformin treatment

The normal dose of metformin is 500 mg three times daily, whether it is given as first-line therapy or to clomifene-resistant women with PCOS. It is generally recommended for women to take the drug before meals and increase the dose slowly from once daily, by a rise of 500 mg/day per week, to reduce adverse gastrointestinal effects. Additionally, a dosage of 850 mg twice daily can be recommended to increase compliance [46].

## Conclusions

From the present review, it could be concluded that the most common cause that leads to anovulatory infertility is PCOS. It is a condition that is associated with IR in peripheral tissues, hyperandrogenism, and hyperinsulinemia. The rise in intraovarian androgens is thought to play an important role in the anovulatory cycle. Oral insulin-sensitizing agents such as metformin may act by decreasing the peripheral resistance to plasma insulin and hence cause ovulation to resume, restore metabolic and hormonal derangements, and improve fertility in PCOS patients. Therefore, metformin should be seriously considered as the most suitable first-line treatment for anovulatory infertile non-obese patients with PCOS and in patients resistant to clomiphene alone (when clomiphene is used as a first line of treatment). Metformin was also a useful treatment in reducing the risk of ovarian hyperstimulation in women with PCOS undergoing in vitro fertilization. The long-term utilization of metformin remains uncertain when considering the prevention of distant PCOS complications. Consequently, substantial research efforts are imperative before making a conclusive decision on this matter.
